# Prediction During Within- and Cross-Language Phrase Comprehension: Evidence from Eye-Tracking

**DOI:** 10.5334/joc.518

**Published:** 2026-09-21

**Authors:** Jing Tong, Tanja C. Roembke, Wolfgang Scharke, Iring Koch, Andrea M. Philipp

**Affiliations:** 1 Institute of Psychology, RWTH Aachen University, Jaegerstr. 17-19, 52066 Aachen, Germany

**Keywords:** prediction, language processing, bilingualism, activation, inhibition, classifier congruency, visual world paradigm, eye movements

## Abstract

Language comprehension involves actively predicting upcoming words, but it remains unclear how the brain responds when predictions are disconfirmed. In the present study, we examined the effect of prediction disconfirmation by measuring eye movements in two visual-world eye-tracking experiments. Additionally, we explored such processing within one language as well as across a bilingual’s two languages to explore the question of whether the effect of prediction disconfirmation is language-specific or language-independent. In a within-language and a cross-language experiment, Chinese-English bilinguals heard a classifier-noun phrase (e.g., “a grain of rice”) in which the classifier was always presented in Mandarin Chinese, their L1, and selected a picture corresponding to the presented noun from a set of three pictures displayed on the screen. Across trials, the congruency of the classifier-noun phrase was manipulated: the noun could be congruent (e.g., “a grain of rice”) or incongruent (e.g., “a grain of furniture”) with the classifier. Experiment 1 (N = 40) used the same language (participant’s L1, Chinese) for classifier and noun, whereas Experiment 2 (N = 40) used different languages (Chinese for the classifier; but participants’ L2, English, for the noun). The results revealed temporal patterns of eye movements that may reflect residual activation of representations referring to disconfirmed predictions shortly after disconfirmation, followed by a reduction in their influence that may be related to inhibitory processing. Together, these findings suggest similar temporal patterns of eye movements following prediction disconfirmation in within-language and cross-language contexts, pointing to a dynamic and language-independent trajectory in the processing of lexical representations affected by prediction disconfirmation.

## Introduction

Language comprehension unfolds dynamically over time, continuously updating representations as new input becomes available. Prediction, conceptualized as the pre-activation of lexical representations derived from prior semantic context, is typically engaged during language comprehension (DeLong & Kutas, 2020; Grisoni, Boux & Pulvermüller, 2024). Research on prediction during language comprehension has been accumulating over recent years (e.g., Altmann & Mirković, 2009; Foucart et al., 2015; Martin et al., 2018; Chun & Kaan, 2019; Lelonkiewicz et al., 2021; Kim et al., 2023; Ness & Meltzer-Asscher, 2018; Pickering & Gambi, 2018; Rommers & Federmeier, 2018; Ryskin & Nieuwland, 2023; but see Bannon et al., 2025; Kaan & Grüter, 2021, for reports of no reliable prediction effects). In general, these studies demonstrate that prediction is reliably engaged in highly constraining contexts, in which the semantic context strongly biases the set of possible upcoming words and makes a specific continuation highly predictable.

For example, previous research has established that words presented in highly predictable contexts can be processed more rapidly, as evidenced by faster lexical decisions (Fischler & Bloom, 1979) and shorter reading times during sentence reading (Ehrlich & Rayner, 1981). Further, electrophysiological studies also found that event-related potentials (ERPs) were influenced by sentence predictability (DeLong & Kutas, 2020; Grisoni, Boux & Pulvermüller, 2024; Grisoni, Miller & Pulvermüller, 2017; Grisoni, Tomasello & Pulvermüller, 2021; Ness & Meltzer-Asscher, 2018). For example, Grisoni, Boux, and Pulvermüller (2024) found that the prediction potential, defined as the mean ERP amplitude during the 200 ms before the onset of the target noun, was larger when participants heard a high-constraint sentence with a predictable final word than when they heard a low-constraint sentence with an unpredictable final word, suggesting that prediction is engaged in a highly predictable context.

In addition to the above-mentioned studies investigating prediction during native language (L1) processing in monolingual speakers, other studies have examined whether predictive processing similarly occurs in a second language (L2) learned later in life. Some studies reported that participants did not generate predictions based on prior semantic context during L2 comprehension, suggested by the absence of a reduction of the N400 ERP component in predictable compared to unpredictable contexts (Ito et al., 2017; Martin et al., 2013). Yet, other studies provided contrary findings, demonstrating that predictions were also made during L2 comprehension (Chun & Kaan, 2019; Dijkgraaf et al. 2017; Foucart, Martin et al., 2014; Foucart, Ruiz-Tada & Costa, 2016). This inconsistent data pattern across studies could be due to a weaker predictive ability in L2 (Martin et al., 2013) or to slower L2 processing (Dijkgraaf et al., 2017; Momenian et al., 2024), suggesting language-specific differences between native and non-native language processing during prediction. However, the failure to observe a prediction effect in the L2 may alternatively result from the use of indices that do not directly capture predictive processing (cf. Ito et al., 2017; Martin et al., 2013). For example, Ito and colleagues (2017) proposed that the N400 effect may reflect an integration process combining top-down contextual information with bottom-up activation, rather than serving as a direct measure of prediction.

Finally, there is evidence that prediction can interact with language switching (also referred to as code-switching). For example, Liao and Chan (2016) explored semantic predictability and language switching in auditory sentence comprehension in an EEG study. In their study, the last word of a sentence was either presented in the same or in a different language than the rest of the sentence. Additionally, the predictability of this last word was manipulated by using words that had a high vs. low predictability in the specific sentence context. The EEG data showed an interaction between language switching and predictability, such that the effect of predictability was stronger during language switching than during language repetition. However, only in the first 250 to 350 ms after target word onset.

Taken together, previous studies suggest a prediction effect in both within-language experiments (mainly when using L1, e.g., Grisoni, Boux & Pulvermüller, 2024) and in cross-language experiments (e.g., Liao & Chan, 2016). In the present study, we explored the role of prediction both in a within-language and a cross-language experiment to answer the question of whether prediction processes are specific to a monolingual situation or whether they are language-unspecific and show a comparable pattern within- and cross-language. More specifically, this study focused on language processing after a disconfirmed prediction.

In everyday language comprehension, not all incoming input confirms our prediction. When predicted words are disconfirmed, two possible outcomes may occur. One possibility is that the predicted word is actively inhibited (DeLong & Kutas, 2020; Kim et al., 2023; Kutas, 1993; Ness & Meltzer-Asscher, 2018; Sánchez-Meléndez et al., 2024). For example, a study by Kim et al. (2023) found that recognizing a prediction-matching word ‘knife’ in a lexical decision task was slower after hearing ‘Father carved the turkey with a path’ (i.e., sentence with prediction-violated final word), compared with hearing ‘Father carved the turkey with a’ (i.e., sentence without final word). This suggests that the prediction-matching word ‘knife’ had to be inhibited to be able to comprehend the sentence ‘Father carved the turkey with a path’.

However, another possibility is that the predicted word remains activated. This possibility has been supported by the study of Rommers and Federmeier (2018). In their study, participants read low constrained sentences in which the critical sentence-final word was either a word presented two sentences earlier, a word that had been predicted but subsequently disconfirmed in a highly constraining context two sentences earlier, or an entirely new word that had not been previously encountered. Their results showed that, relative to new words, both previously seen words and previously expected but disconfirmed words elicited a reduced N400, suggesting that disconfirmed predictions remained activated (i.e., lingered) in memory.

The experimental paradigms used to support the two opposing accounts (i.e., inhibition vs. activation) share a key methodological feature. In both cases, participants first encounter a highly constraining context that strongly predicts a particular word. This prediction is then disconfirmed by presenting an unexpected alternative, after which the originally predicted word is re-presented at a later point. Researchers infer inhibition or activation from participants’ responses to this later re-presentation of the previously predicted word. Consequently, the conclusions drawn from these studies are necessarily indirect, as they rely on the assumption that responses to the later re-presentation reflect the cognitive processes towards the predicted word at the moment when the prediction was initially disconfirmed, rather than providing direct evidence of whether the word was inhibited or remained activated during online language processing. Further, it remains an open empirical question whether inhibition and activation accounts should be regarded as mutually exclusive explanations, or whether they can coexist, potentially reflecting different temporal stages of processing, a possibility that has not yet been directly examined in previous experimental designs (e.g., Kim et al., 2023; Rommers & Federmeier, 2018). The present study was aimed to address this issue by examining whether (and for how long) the representation referring to a disconfirmed prediction remains activated in both within-language and cross-language contexts, and whether the level of activation differs between these contexts. If eye movement patterns associated with residual activation are observed, this would suggest that the activation plays a role in processing lexical representations corresponding to disconfirmed predictions. In contrast, if no such activation is detected, this could potentially lend support to the inhibition account. We use the term prediction disconfirmation to refer to the process by which a previously generated prediction is shown to be incorrect. The resulting effect on the associated lexical representation, such as its continued maintenance or inhibition, is subsequently referred to as the effect of prediction disconfirmation.

To address this question, the present study adopted the visual world paradigm (VWP). This paradigm has been extensively validated as a sensitive online measure of lexical processing, capable of indexing the activation strength of lexical representations (e.g., Allopenna et al., 1998; Huettig et al., 2011; Roembke & McMurray, 2016; Roembke & McMurray, 2025). In this paradigm, participants’ eye movements are recorded while they view a set of visual objects and listen to an auditory target word. Critically, a robust finding is that participants’ visual attention is drawn not only to the visual object referring to the auditory target but also to visual competitor objects semantically related to the auditory target (e.g., Huettig & Altmann, 2005; Yee & Sedivy, 2006). Such fixation patterns provide evidence for the lexical co-activation of the target noun and its competitors during real-time language comprehension. Accordingly, the VWP offers a suitable method for examining whether the representation referring to a disconfirmed prediction remains activated (at least for some time).

To manipulate predictability, the present study used classifier congruency. Both Chinese and English have a rich system of classifiers or classifier-like expressions (Allan, 1977, 1978) and in both languages, classifier convey semantic and grammatical information about the following noun (cf. Grüter et al., 2020). For example, the classifier “a grain of” provides information about the quantity and type of entity denoted by the noun. It combines naturally with ‘rice’ but not with ‘furniture’. More generally, classifier–noun combinations in both Chinese and English are not arbitrary: certain classifiers are conventionally associated with specific nouns, and certain nouns typically co-occur with particular classifiers (Lehrer, 1986). Thus, for classifier congruency, our primary interest lies in whether a classifier can be meaningfully and naturally associated with a given noun. Accordingly, we refer to the contrast between ‘a grain of rice’ and ‘a grain of furniture’ as semantic classifier congruence versus semantic classifier incongruence. Previous studies have shown better performance in a classifier congruent condition (e.g., ‘a grain of rice’) than in a classifier incongruent condition (e.g., ‘a grain of furniture’), indicating a classifier congruency effect (cf. Tong et al., 2025a, 2025b; see also Huang & Schiller, 2021; Wang et al., 2019; Zhang & Liu, 2009, albeit classifier congruence was operationalized in a slightly different way in these studies). In addition, two eye-tracking studies in within-language comprehension found that participants looked more to the semantically congruent noun after comprehending the classifier (Huettig et al., 2010; Mitsugi, 2020). This finding clearly indicates that people activate predictions during classifier processing (see also Grüter et al., 2020).

In our previous study (Tong et al., 2025b), we manipulated classifier congruency to examine predictability and investigated predictive processing in within-language and cross-language production. Participants were first presented with a classifier auditorily in their L1 (Mandarin Chinese) or their L2 (English) and then a picture was shown, whose name could be congruent or incongruent with the classifier (e.g., ‘a crew of firemen’ vs. ‘a crew of rain’). Chinese-English participants were asked to name the picture in the language indicated by a color frame. Results showed a within-language classifier congruency effect but no cross-language effect. More specifically, after participants heard a classifier, a congruent picture was named faster than an incongruent picture when classifier presentation and naming response were in the same language (e.g., both in the L1). However, a classifier congruency effect was not observed when the picture was named in a language other than that of the classifier. That is, a prediction effect was absent when classifier and response language differed, regardless of the language direction (i.e., L1 classifier-L2 response or L2 classifier-L1 response). Although these findings appear to suggest an absence of a prediction effect in a cross-language context, this outcome may be attributable to a task-specific factor. Specifically, we speculated that the absence of a prediction effect could be due to prediction being language-specific in a language-production task the word has to be produced in the correct language, so that both the concept and the language play a role. In contrast, in the present study, a language-comprehension instead of a language-production task was used, where participants were able to generate a language-unspecific prediction.

In addition, although previous studies have demonstrated a prediction effect during L2 comprehension (e.g., Chun & Kaan, 2019; Dijkgraaf et al., 2017; Foucart, Martin et al., 2014; Foucart, Ruiz-Tada & Costa, 2016), these studies have primarily focused on within-language contexts (i.e., L2 only). By contrast, our previous study examined prediction in a cross-language context and found that the language direction of the classifier and the response did not affect the results. Thus, we chose to use only one language direction in the present study. We selected L1 as the classifier language, as larger prediction effects are generally expected in L1 compared to L2 (see Tong et al., 2025a, 2025b).

## Current study

This study aimed to investigate whether a disconfirmed prediction remains activated in within-language and cross-language contexts and whether the strength and time course of activation differed between these two contexts. To this end, we adopted the VWP to investigate disconfirmed predictions during comprehension. Chinese-English bilinguals heard a phrase consisting of a classifier and a noun and were asked to click on the picture corresponding to the noun. Note that the picture corresponding to the auditory target noun was referred to as the target picture, whereas any picture that did not correspond to the auditory target noun was defined as a non-target picture. Next to the reaction time (RT) and error rate for the target response (i.e., not clicking on the target picture), we also measured eye movements to pictures that were (in-)congruent to the classifier as a measure of in-the-moment processing.

A visual target picture was always presented corresponding to the auditory target noun. Given that the relationship between the classifier and the auditory target noun could be either congruent or incongruent, the corresponding target picture was likewise classified as congruent or incongruent with the classifier (i.e. classifier-noun congruence always also refers to classifier-target picture congruence). Additionally, the two non-target pictures in each trial also could be congruent or incongruent to the classifier, which we refer to as congruent or incongruent non-target pictures. Trials fell into three categories (see Table 1): 1) One picture was congruent to the classifier, whereas two were incongruent. 2) Two pictures were congruent to the classifier, whereas one was incongruent. 3) Additionally, as a control trial, all three pictures were incongruent to the classifier (all trials in the third category were considered filler trials; see Methods for details). In all three trial types, any picture could serve as the target picture, meaning that the classifier phrase could be either congruent or incongruent with the target picture, defined as the picture corresponding to the auditory target word. This manipulation prevented participants from forming strong expectations about which picture would ultimately be selected and discouraged them from adopting the strategy of always attending to the classifier-congruent picture. Consequently, if participants nevertheless showed greater anticipatory looking toward a classifier-congruent picture than toward an incongruent picture, this would provide evidence that they generated predictions during classifier comprehension rather than relying on task-specific expectations.

**Table 1:** Classifier-picture set types in Experiment 1 and Experiment 2 with one example classifier–noun phrase for each type. In each set, either picture could serve as the target picture corresponding to the auditory target word.


CLASSIFIER-PICTURE SET	CLASSIFIER	PICTURE 1	PICTURE 2	PICTURE 3

One-congruent		**Congruent**	Incongruent	Incongruent

	a grain of	**rice**	furniture	newspaper

	**一粒**	**米**	**家具**	**报纸**

	yī lì	**mǐ**	jiā jù	bào zhǐ

Two-congruent		**Congruent**	**Congruent**	Incongruent

	a grain of	**wheat**	**corn**	donut

	**一粒**	**麦子**	**玉米**	**甜甜圈**

	yī lì	**mài zi**	**yù mǐ**	tián tián quān

None-congruent		Incongruent	Incongruent	Incongruent

	a grain of	clothes	cigarettes	plate

	**一粒**	**衣服**	**烟**	**盘子**

	yī lì	yī fú	yān	pán zi


The inclusion of displays containing one versus two classifier-congruent pictures served a similar design purpose by increasing variability across trials. Thus, this manipulation was implemented to reduce task-specific expectations rather than for testing the effect of the number of congruent pictures. Accordingly, we had no theoretical reason to expect differences in behavioral responses or eye movements between these two display types. Therefore, the analyses of RT, accuracy, and eye-movement combined the two display types that contained both a congruent and an incongruent non-target picture: (i) trials with one classifier-congruent picture in which the auditory noun and the corresponding visual target picture were incongruent to the classifier, and (ii) trials with two classifier-congruent pictures in which the auditory noun and the corresponding visual target picture were congruent to the classifier. More specifically, our critical comparison in the eye-movement analyses concerned the processing of a classifier-congruent non-target picture (reflecting a disconfirmed prediction) relative to a classifier-incongruent non-target picture (reflecting a non-predicted concept) following noun onset. In RT and error analyses, the same trials were used to ensure consistency across the analyses. However, in these analyses we focused on the congruent and incongruent targets (i.e., RT and error rate for the response on the target picture). These design choices also ensured that half of the trials had a congruent and half of the trials had an incongruent target picture.

In Experiment 1, the classifier and noun language was the same (i.e., Mandarin Chinese), whereas in Experiment 2, the classifier and noun languages were different (the classifier was always presented in the L1, Mandarin Chinese, and the noun was presented in the L2, English). Based on the research by Rommers and Federmeier (2018), which demonstrated the activation of a disconfirmed prediction, together with findings from our earlier studies (Tong et al., 2025a, 2025b) showing a prediction effect arising from classifier comprehension, we had three expectations concerning the outcomes of our two experiments.

Firstly, based on our previous findings showing a prediction effect during classifier comprehension (Tong et al., 2025a, 2025b), we expected that in both experiments participants would click on the target picture faster when the auditory noun and the corresponding visual target picture were congruent to the classifier than when they were incongruent.

Secondly, we expected that in both experiments, after hearing the noun, a predicted word/concept might remain activated despite not being the target (Rommers & Federmeier, 2018). Such activation should result in a higher looking proportion for the congruent non-target picture than for the incongruent non-target picture.

Finally, given that a previous study (Liao & Chan, 2016) reported an interaction between semantic predictability and language switching, we were interested to see whether the above-mentioned congruency effect for non-target pictures differed between the two experiments.

## Experiment 1: Within-Language Prediction

### Method

#### Participants

Forty participants studying at RWTH Aachen University took part in this in-lab study (22 females, 17 males, 1 person did not report their gender; 40 right-handed; age: M = 25.15 years, SD = 4.34 years). Mandarin Chinese was their native language (L1). All procedures in our study were conducted in accordance with the protocol approved by the Ethics Committee of the Faculty of Arts and Humanities at RWTH Aachen University. All participants signed written informed consent and data protection.

#### Materials and apparatus

For the experimental trials, 26 classifiers and 78 nouns/pictures were selected as stimulus material by the experimenters (see Appendix A for an English version of the Chinese classifiers and nouns).

Each classifier was used in three different sets. In each set, a classifier was always combined with three different visual pictures and each of these pictures was once the visual target picture – indicated by the auditory noun. That is, each set (classifier + three pictures) was used in three trials in which the classifier was combined with three different nouns, resulting in three different classifier-noun phrases (and three different target pictures correspondingly). Additionally, for each classifier, one set consisted of three pictures whose corresponding concept/word were incongruent with the classifier. The other two sets were either one-congruent picture sets or two-congruent-pictures sets (see Table 1 for an overview of the three set type categories). The visual target picture could either be congruent or incongruent, with congruence being defined as whether the corresponding classifier-noun combination would be commonly used in daily life (e.g., ‘a grain of rice’ being congruent vs. ‘a grain of furniture’ being incongruent). Across all classifiers and sets, the number of one-congruent-picture and two-congruent-pictures sets was the same. However, some classifiers only occurred in one-congruent-picture sets, while others occurred only in two-congruent pictures sets or in both kinds of sets. This distribution was based on the number of congruent nouns that could be combined with each classifier. For example, ‘a pair of’ can be combined with a large number of nouns in a meaningful and naturally-used way and, thus, occurred twice in a two-congruent-pictures set, whereas ‘a drop of’ has less congruent nouns so that it occurred twice in a one-congruent-picture set.

Each of the 78 nouns/pictures also occurred in three different sets but was never combined with the same classifier or another noun/picture more than once. To prevent that one of the three nouns/pictures in each set was more salient than the others because of its semantic relationship with the remaining items, the three pictures within each set were either all semantically related (e.g., wheat, corn, and donut are all food items) or all relatively unrelated (e.g., rice, furniture, and newspaper). This design avoided situations in which one noun/picture was semantically distinct from the other two, thereby reducing potential attentional biases unrelated to the experimental manipulation. Additionally, the three nouns did not phonologically overlap in Chinese (and in English, which is important for Experiment 2). A separate group of 26 English native speakers were asked to rate English classifier-noun phrases on a five-point scale (1 = very uncommon, 5 = very common). A paired-sample t-test showed that there was a significant difference between congruent and incongruent classifier-noun combinations (1.57 [incongruent] vs. 4.66 [congruent]), *t*(25) = –40.13, *p* < .001), indicating that the classifier-noun phrases used as congruent or incongruent phrases in the experiment clearly differed in terms of their congruency rating.

Corresponding to the 78 nouns, we selected 78 real-life pictures (350 × 350 pixels, corresponding to approximately 6.17° by 6.17° of visual angle). Fifty-five of them were taken from the LinguaPix database (Krautz & Keuleers, 2022) and the remaining twenty-three pictures were adapted from licensed online images. To make sure the adapted online pictures corresponded accurately to the nouns, we first selected three different pictures for each of the twenty-three nouns. A separate group of twenty native Chinese speakers was subsequently asked to select the best-matching picture from the three options. The pictures that were selected by the majority of the participants were then used as stimulus material.

All Chinese classifiers and nouns were presented auditorily and were generated by a text-to-speech system in Google Cloud to generate realistic-sounding human voices (Format: wav; name: cmn-CN-WaveNet-B; speed rate: normal; sampling rate: 44100 Hz). Despite the varying durations of each classifier audio file (M = 762 ms, SD = 38 ms), we kept a constant interval of 1500 ms between the onset of the classifier and the onset of the target noun. That is, there was a silent interval of variable duration before the target-noun onset.

The experiment was programmed and executed using PsychoPy (Peirce et al., 2019), version 2021.2.3. Eye movements were recorded at a sample rate of 1000 Hz with an Eyelink 1000 Plus system (SR Research Ltd, Ottawa, Ontario, Canada), with participants’ heads stabilized by a desk-mounted chin and forehead rest. Only the right eye of each participant was tracked. The picture-clicking reaction time (RT; measured as the time from the onset of the target noun to the timestamp that participant clicked the mouse button after moving the cursor to the correct picture) and accuracy after the target nouns onset was recorded via PsychoPy.

#### Procedure

Before the experiment, participants were familiarized with all the pictures. For this familiarization, all pictures and picture names were presented in a PowerPoint presentation (i.e., each slide showed one picture and the corresponding picture noun in Chinese). Then participants sat in front of a 24” TFT LCD screen (BenQ XL2411P) with a resolution of 1920 × 1080 pixels positioned at a distance of 90 cm from their eyes. After reading the task instructions (in Chinese) displayed on the screen, a nine-point calibration procedure for the eye-tracker was conducted. Participants began with a practice block consisting of 12 trials to ensure comprehension of the task instructions (both classifier and nouns/pictures used in the practice block differed from those in the actual experiment). A drift correction was conducted at the beginning of each trial (both practice and experimental trials).

**Figure 1:**
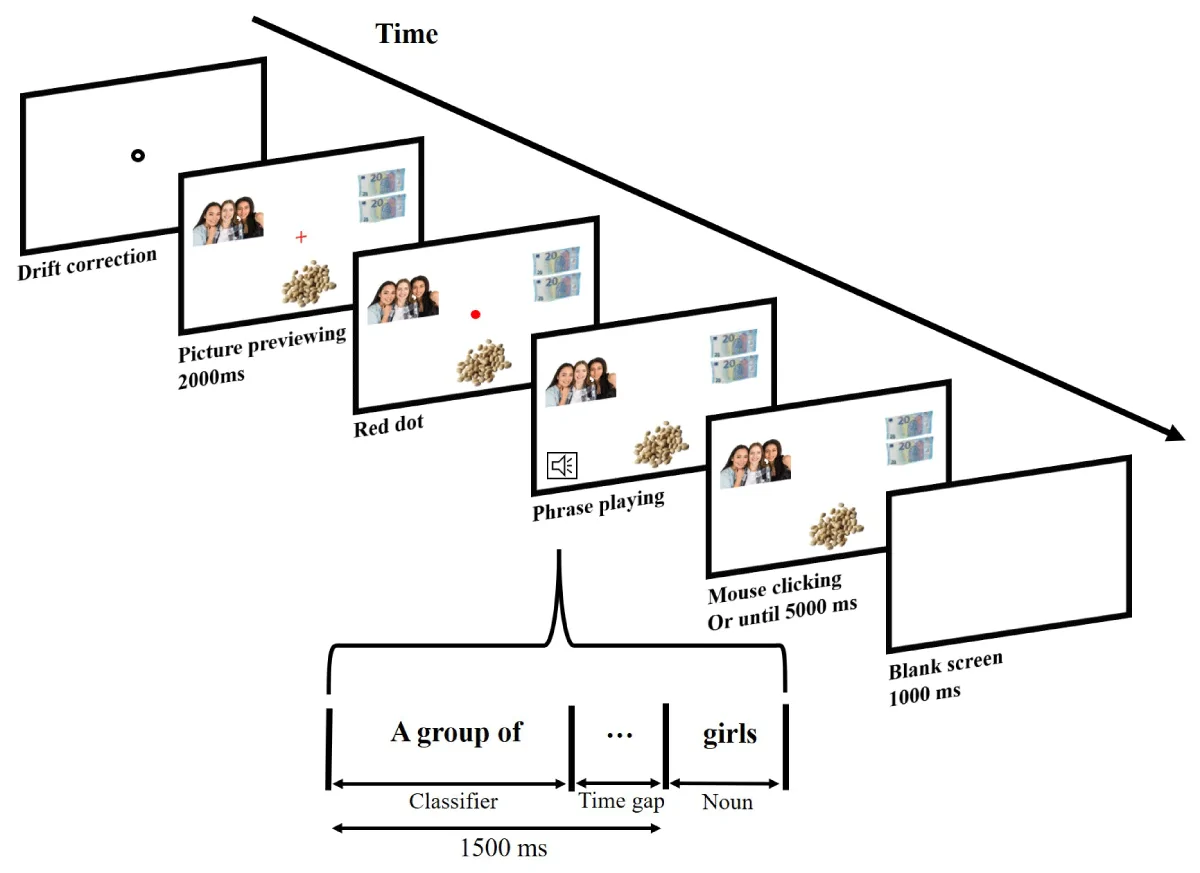
Illustration of procedure in a trial and the time window during phrase playing. Note. Due to variations in the duration of different classifiers, we ensured that the interval between the onset of the classifier and the onset of the target-noun was consistently 1500 ms by inserting a variable time gap in between. All the pictures were selected from the LinguaPix database (Krautz & Keuleers, 2022) or were adapted from licensed online images. In this figure, the example stimuli are shown in English; however, the classifier was always presented in Chinese, and the noun could be either in Chinese (Experiment 1) or in English (Experiment 2).

In each trial (see Figure 1), three pictures appeared on the screen, with either two pictures at the bottom and one on the top, or vice versa (cf. Roembke & McMurray, 2016). The assignment of the pictures to the locations was randomized in each trial using PsychoPy (Peirce et al., 2019). After previewing the pictures for 2000 ms, a red dot appeared in the center of the screen. Participants were asked to click with the computer mouse on the red dot if they completed the picture previewing (without time limits). Upon clicking on the red dot, it disappeared and a Chinese classifier-noun phrase was presented auditorily while the three pictures still remained on the screen. Participants were asked to click on the target picture, which was the picture that corresponds to the noun in the auditory classifier-noun phrase. After clicking, or when participants failed to respond within 5000 ms, the pictures disappeared. Then, the screen turned blank for 1000 ms before the next trial started.

#### Data analysis

**RT analysis**. Error trials, defined as incorrect picture selections or no response, as well as all trials with response times (RT) beyond ± 3 standard deviations from the mean RT for each participant were excluded from the RT analysis. Consequently, 1.61% of all trials were excluded. A paired-samples t-test was conducted to compare trials in which the target noun and thus also the target picture were congruent vs. incongruent to the classifier (i.e., classifier-noun congruency; also note that, in the RT analysis, congruency refers to the relationship between the classifier and the target noun. In the looking-proportion analyses, however, congruency refers to the relationship between the classifier and both the target and non-target pictures.). To ensure consistency with the looking-proportion analyses, we included only trials with one congruent picture in which the target was the incongruent picture and trials with two congruent pictures in which the target was a congruent picture (see the Looking Proportions Analyses section below for the rationale).

**Accuracy analysis**. Since accuracy was close to ceiling (99.59 % correct responses), it was not subjected to further analysis.

**Looking proportions analyses**. We used the VWPre package (version 1.2.3; Porretta et al., 2018) for data preprocessing and the clusterperm package (Barr, n.d.) to analyze the data. The data were binned into 20-ms intervals, and the looking proportion for each picture was defined as the proportion of samples with a gaze position on that specific picture within each interval. Each trial with more than 25% track loss caused by eye blinks was excluded from the analysis. In addition, trials with an incorrect response, or no response were also excluded. In total, 0.41% of all trials were excluded from looking proportion analyses. The time window of interest was defined as the interval from the start of classifier presentation to the average end of the noun presentation. This interval lasted 1500 ms for classifier presentation and 780 ms for noun presentation,^1^ resulting in a total duration of 2280 ms.

As our second and third hypotheses referred to disconfirmed predictions, only the looking proportions for non-targets were of interest. To evaluate the experimental hypotheses, the analysis focused exclusively on the looking proportions toward the congruent and incongruent non-target pictures. The trials included in the analysis were trials with one congruent picture in which the target was an incongruent picture and trials with two congruent pictures in which the target was a congruent picture, that is, these trials always contained a congruent and an incongruent non-target picture, resulting in equal numbers of non-targets of each type. This also ensures a balanced comparison in which half of the classifiers were paired with a noun semantically congruent with the classifier, whereas the other half were paired with a semantically incongruent noun.

After aggregating the looking proportions across items for each of the two picture types per participant per interval, we performed cluster-based permutation analysis on the looking proportions in the time window of interest. The procedure for conducting the cluster-based permutation analysis was as follows (Barr, 2022): Firstly, at each interval, we ran an ANOVA and extracted the corresponding test statistic (F-value) and p-value. Consecutive intervals with p-values below 0.05 were grouped into clusters, and for each cluster we computed the cluster-mass statistic. Secondly, to generate the null-hypothesis distribution, we randomly permuted picture type labels (i.e., congruent non-target and incongruent non-target picture) within subjects 1000 times and recomputed the largest cluster-mass statistic for each permuted data set to create the null hypothesis distribution. Finally, each cluster from the original data was compared against this permutation-based null distribution to obtain Monte Carlo p-values.

### Results

#### Reaction time

Participants selected the target picture significantly faster in trials where the auditory noun and the visual target picture were congruent with the classifier than in trials where noun/target picture were incongruent with the classifier (1129 ms vs. 1187 ms). A paired-samples t-test revealed that this classifier congruency effect of 58 ms was significant (*t* = –6.44, *p* < .001).

#### Looking proportions

**Figure 2:**
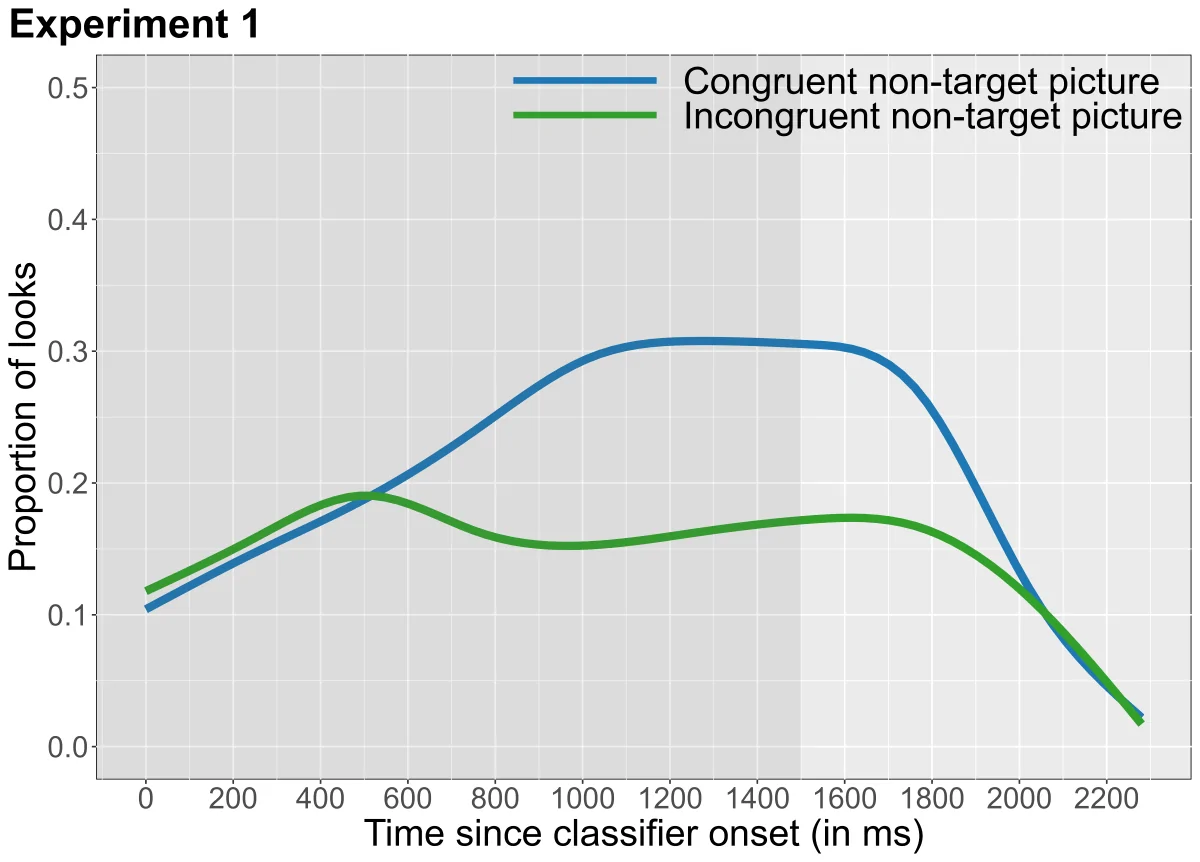
Proportion of looks in Experiment 1. Note: The first dark gray time window (0–1500 ms) represents the time window between the classifier onset and target-noun onset. The second light grey time window (1500–2280 ms) represents the time window after noun onset.

Cluster-based permutation analysis showed a significant cluster from 620 ms to 1920 ms (cluster mass statistic = 1574.49, *p* < 0.01), showing higher looking proportions for a congruent non-target picture as compared to an incongruent non-target picture (see Figure 2). This significant cluster indicated that the congruent picture attracted greater attention during the classifier presentation period (620–1500 ms), as well as during the early phase of the noun presentation (1500–1920 ms), when it had become irrelevant.

### Discussion

In Experiment 1, consistent with the findings from our previous studies (Tong et al., 2025a, 2025b), we found a classifier congruency effect for RTs in participants’ L1 (Mandarin Chinese). That is, participants were faster to click on a classifier congruent target picture than on a classifier incongruent target picture.The novel finding refers to the eye-tracking data. Participants directed more looks toward the congruent picture than the incongruent picture during classifier presentation, indicating that they engaged in semantic prediction during within-language classifier comprehension. This pattern persisted into the early stage of noun presentation even after the congruent picture had become irrelevant, with the congruent non-target picture continuing to attract more fixations than the incongruent non-target picture. This fixation pattern is compatible with the possibility that the representation of predicted word/concept remained relatively more active during this early stage of noun processing. Following this residual activation of the predicted but disconfirmed word/concept during the early stage of noun presentation, no additional significant clusters were observed. This suggests that participants allocated comparable levels of attention to both the congruent and incongruent non-target pictures at that time point.

## Experiment 2: Cross-Languages Prediction

### Method

#### Participants

Forty participants studying at RWTH Aachen University took part in this in-lab study (19 females, 19 males, 2 persons did not report their gender; 40 right-handed; age: M = 23.93 years, SD = 3.34 years). Mandarin Chinese was their native language (L1) and English was their second language (L2). They completed a questionnaire about the age of L2 acquisition (age: M = 6.88 years, SD = 2.44 years), and their self-rated language skills for English compared to those of a native English speaker (7-point scale: 1 = “quite poor”, 7 = “highly proficient”; writing: M = 4.50, SD = 1.30; speaking: M = 4.45, SD = 1.28; reading: M = 4.95, SD = 1.16; M = 4.58, SD = 1.20), which indicated that they rated themselves as less-fluent English speakers compared with native English speakers.

#### Materials and apparatus

The materials and apparatus were identical to those used in Experiment 1 except that the auditory target nouns were presented in English. The audio files of English nouns were generated by a text-to-speech system in Google Cloud (Format: wav; name: en-GB-WaveNet-B; speed rate: normal; sampling rate: 44100 Hz). We intentionally selected this voice type in the text-to-speech system to ensure it resembled the sound of the Chinese classifier, so that participants had the impression of a coherent classifier-language phrase despite the language switch.

#### Procedure and design

The procedure and the design were the same as in Experiment 1, with the exception (as mentioned previously) that target nouns were always presented in English. Classifier presentation remained always in Mandarin Chinese.

#### Data analysis

**RT analysis**. Error trials, defined as incorrect picture selections or no response, as well as all trials with RTs beyond ± 3 standard deviations from the mean RT for each participant were excluded from the RT analysis as in Experiment 1. Consequently, 2.45% of the total trials were excluded.

**Accuracy analysis**. Since accuracy was close to ceiling (99.35 %), it was again not subjected to further analysis.

**Looking proportion analyses**. We analyzed the looking proportion following the same procedure as in Experiment 1. Trials with over 25% track loss due to eye blinks, incorrect response, or no response were excluded, resulting in 0.65% of all trials being omitted from the looking proportion analyses. The time window of interest was defined as the interval between the start of the auditory Chinese classifier presentation and the end of the auditory English noun presentation. This included 1500 ms for the classifier presentation and 780 ms for the noun presentation, resulting in a total time window of 0 to 2280 ms.^2^

### Results

#### Reaction time

Participants selected the target picture significantly faster in trials where the auditory noun and its corresponding visual target picture were congruent with the classifier than in trials where the noun/target picture were was incongruent with the classifier (1150 ms vs. 1218 ms). A paired-samples t-test revealed that this classifier congruency effect of 68 ms was significant (*t* = –6.85, *p* < .001).

#### Looking proportions

**Figure 3:**
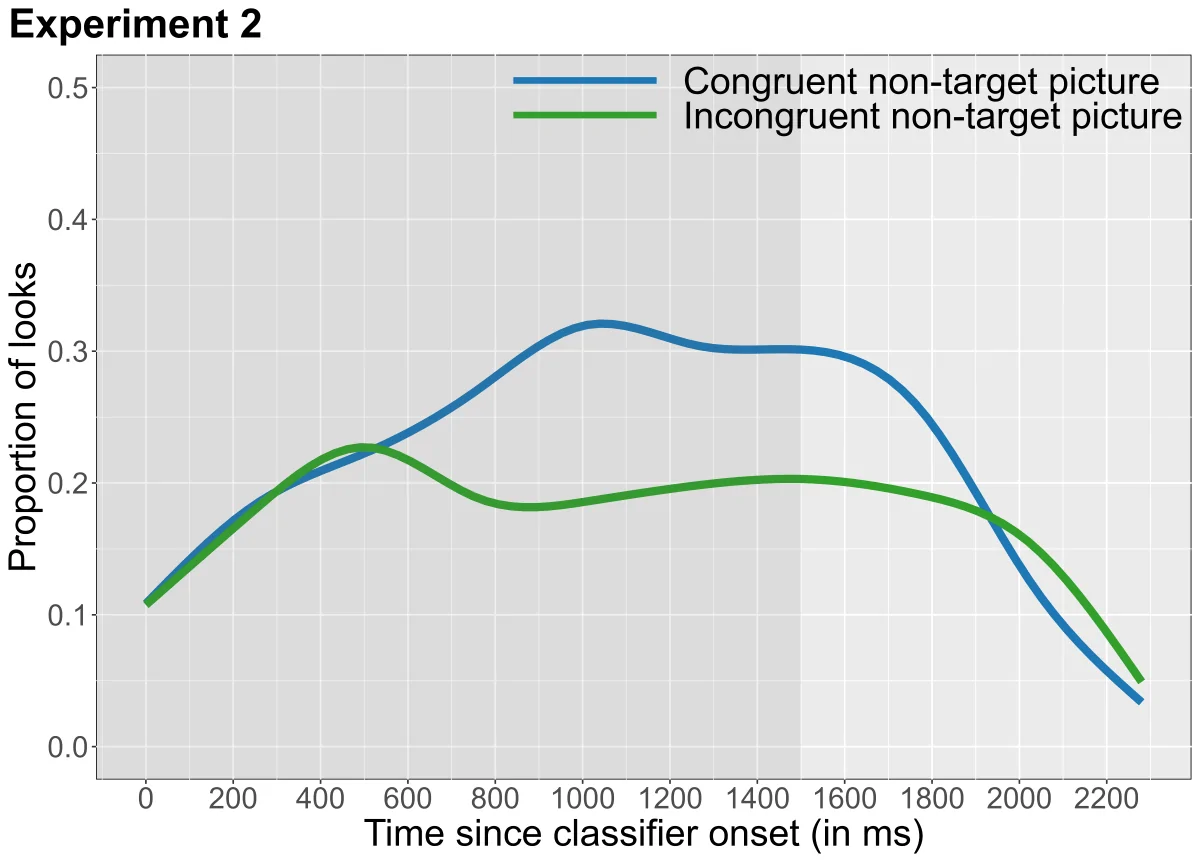
Proportion of looks in Experiment 2. Note: The first dark gray time window (0–1500 ms) represents the time window between the classifier onset and target-noun onset. The second light grey time window (1500–228 ms) represents the time window after noun onset.

Cluster-based permutation analysis showed a significant cluster from 660 ms to 1800 ms (cluster mass statistic = 1091.19, *p* < 0.01), showing higher looking proportions for congruent non-target pictures as compared to incongruent non-target pictures (see Figure 3). This significant cluster suggested that the congruent picture received more attention, beginning in the classifier presentation window (660–1500 ms) and extending into the early noun presentation window (1500–1800 ms), when it had become irrelevant. A further significant cluster between 2000 ms and 2260 ms revealed a reversed pattern (cluster mass statistic = 210.31, *p* = .04), with participants looking more to the incongruent non-target picture than to the congruent non-target picture (see Figure 3).

### Discussion

As in Experiment 1, we found a classifier congruency effect in the RTs. Additionally, increased looks to the congruent picture compared to the incongruent picture during classifier presentation suggested that participants were making predictions while processing the classifier. That is, participants generated predictions based on their L1, Mandarin Chinese, for auditory target-nouns presented in their L2, English, consistent with cross-language prediction. Again, as in Experiment 1, this fixation advantage continued during the early phase of noun presentation, with the congruent non-target picture attracting more looks than the incongruent non-target picture. One possible interpretation of this fixation pattern is that the predicted representation remained relatively more active shortly after prediction disconfirmation. However, unlike in Experiment 1, the congruent non-target picture subsequently received fewer looks than the incongruent non-target picture. This later fixation pattern may reflect a reduction in the influence of the disconfirmed prediction over time, although the present data do not allow us to determine the underlying cognitive mechanism.

### Between-experiment comparisons

To examine whether the effect of prediction disconfirmation was language-specific or language-independent, we examined the language switch from auditory classifier to auditory target affected RT and looking behavior. To this end, we ran a mixed two-way ANOVA with between-subjects factor experiment type (Experiment 1 vs. Experiment 2) and within-subjects factor classifier–noun congruency (target congruent vs. incongruent to the classifier) as independent variables. It revealed a significant main effect of classifier–noun congruency (*F*(1,78) = 88.29, *p* < .001, *η^2^_P_* = .53), showing a shorter RT when the target noun was congruent to the classifier than when it was incongruent. Yet there was no effect of experiment or an interaction of classifier–noun congruency with experiment (*ps* > .43), suggesting that the observed classifier–noun congruency effect was not affected by the language switching in Experiment 2.

The looking proportions were analyzed using the same procedure as in Experiments 1 and 2, with the exception that the labels in experiment type were randomly assigned to the participants and those in picture type were randomly permuted in each participant in order to generate the null-hypothesis distribution. We included the experiment type labels permutation in order to test the main effect of experiment type and the interaction between experiment type (Experiment 1 vs. Experiment 2) and picture type (i.e., congruent vs. incongruent non-target pictures), see Barr (2022).

Cluster-based permutation analysis showed a significant cluster of experiment type from 2000 ms to 2280 ms (cluster mass statistic = 143.38, *p* = 0.04), showing higher looking proportions in Experiment 2 as in Experiment 1. In addition, a significant cluster of picture type from 620 ms to 1900 ms was found (cluster mass statistic = 2630.29, *p* < .001), showing higher looking proportions for congruent non-target pictures as compared to incongruent non-target pictures. However, the interaction between experiment type and picture type did not yield any significant clusters (cluster mass statistic = 107.45, *p* = .06), suggesting that looking behavior was more or less similar across experiments despite the language switch in Experiment 2.

## General Discussion

The present study investigated whether a disconfirmed prediction remains activated in both within-language and cross-language contexts, and whether the level and temporal pattern of activation differs between these contexts. While Experiment 1 in the present study was conducted in a within-language context (participants’ L1), Experiment 2 was conducted in a cross-language context (participants’ L1, followed by the L2). Both experiments employed the VWP, which allowed us to investigate real-time eye movements following the auditory presentation of a classifier and a noun.

For the RT data in both experiments, we expected a classifier congruency effect, such that participants would click on the target picture faster when the noun was congruent with the classifier than when it was incongruent. This hypothesis was confirmed in both experiments, which is consistent with findings from previous studies (Tong et al., 2025a, 2025b). The observed classifier congruency effect indicates the presence of a prediction effect, both within- and across-language contexts.

The novel contribution of the present study refers to the inclusion of eye-tracking to examine the temporal dynamics of predictive processing in auditory language comprehension. With respect to looking proportions, we focused exclusively on non-target pictures that were either congruent or incongruent to the classifier, but did not correspond to the presented target noun. Given the design of our experiments, specifically the possibility that participants could click on any picture on the screen, the lexical representations corresponding to both the congruent and incongruent non-target pictures are expected to exhibit comparable levels of activation a priori. However, we still found that more looks were directed toward a congruent picture than an incongruent picture in both experiments during the classifier presentation (620 ms – 1500 ms for Experiment 1; 660 ms – 1500 ms for Experiment 2). Consistent with previous eye-tracking studies (Huettig et al., 2010; Mitsugi, 2020), this finding suggests that hearing a classifier activates congruent concepts. That is, participants formed a prediction about the upcoming word (and thus also about the target picture) rather than merely activating the lexical representations associated with all pictures displayed on the screen. This is an important confirmation of word activation based on prediction during comprehension, consistent with previous studies (e.g., Grisoni, Boux & Pulvermüller, 2024; Grisoni, Tomasello & Pulvermüller, 2021).

After the target noun was presented, both the congruent and incongruent non-target pictures became irrelevant. In Experiment 1, at the early stage of the target noun presentation window, higher looking proportions were still found for the congruent non-target picture as compared to the incongruent non-target picture (1500 ms – 1920 ms). This result suggests that participants continued to direct more fixations toward the classifier-congruent non-target picture immediately after auditory noun onset. This fixation pattern is consistent with the predicted word remaining relatively more activated during classifier comprehension and shortly after noun presentation. However, because eye movements are influenced by both lexical processing and oculomotor dynamics (McMurray, 2023), this interpretation should be regarded as tentative. By the end of the auditory target noun presentation time window, looking proportions were comparable for the two non-target pictures in Experiment 1, suggesting that the disconfirmed prediction is not persistently highly activated. Therefore, only the effects observed in the early stage of the noun presentation window align with the findings of Rommers and Federmeier (2018), whereas the effects in the later stage do not. One possible explanation for this pattern is that inhibitory processes contribute to the reduction in visual attention to the disconfirmed prediction following auditory noun presentation. As a result, the disconfirmed prediction may have been inhibited down to the same level as an irrelevant and non-predicted concept (i.e., the incongruent non-target picture) in order to shift visual attention to the picture representing the correct target concept. However, the present data cannot distinguish inhibitory suppression from alternative explanations based on normal fixation dynamics (e.g., activation decay). This ambiguity reflects an inherent limitation of eye-movement measures, as similar fixation patterns can arise from different underlying cognitive mechanisms (McMurray, 2023).

In Experiment 2, during the early target noun presentation window (1500 ms –1800 ms), the congruent non-target picture attracted more looks than the incongruent picture, consistent with the pattern found in Experiment 1. However, during a late noun presentation window (2000 ms – 2260 ms), results showed a reversed data pattern, with lower looking proportions to the congruent non-target picture as compared to the incongruent non-target picture. One interpretation of this reversed fixation pattern is that inhibitory processes reduce the influence of the disconfirmed prediction following noun recognition. Specifically, following disconfirmation, participants looked less often at the congruent non-target picture, which corresponded to the predicted but ultimately incorrect concept, than at the incongruent non-target picture, which did not correspond to the preceding prediction. This pattern is consistent with the possibility that the predicted concept was actively suppressed following disconfirmation, thereby reducing its subsequent activation and, consequently, its visual uptake. We suspect that in a cross-language context, a congruent non-target concept interfered more strongly with the target concept than an incongruent non-target concept did.

Taken together, the results from Experiments 1 and 2 suggest that classifier-congruent non-targets attracted more fixations than incongruent non-targets immediately in the beginning and that this pattern remained for some time after prediction disconfirmation but diminished over time. This temporal fixation pattern is consistent with the possibility that representations associated with predicted but ultimately incorrect words remain relatively more active shortly after disconfirmation before then becoming less prominent during subsequent processing. This pattern at the later stage could potentially reflect the operation of inhibitory processes that serve to suppress the pre-activated (but incorrect) representation following disconfirmation (Kim et al., 2023; Ness & Meltzer-Asscher, 2018; Sánchez-Meléndez et al., 2024). Such inhibition may resolve competition between the disconfirmed prediction and the actual input, ultimately facilitating accurate comprehension. The findings, suggesting a pattern of residual activation followed by the suppression for disconfirmed predictions, represent a novel contribution to the literature, as they provide valuable additional detail on the time-varying nature of post-disconfirmation processing in language comprehension. However, because fixation curves provide an indirect measure of underlying activation and/or inhibition processes and are additionally influenced by oculomotor dynamics (McMurray, 2023), the present findings should be interpreted as compatible with, rather than definitive evidence for, sustained activation and subsequent inhibition.

Regarding the comparison of the two experiments, for RTs, we did not find a main effect of experiment type or the interaction of experiment type and classifier–noun congruency. When comparing looking proportions, we only found a main effect of experiment type with higher looking proportion in Experiment 2 as compared to Experiment 1. Apart from that, no interaction of experiment type and picture type was found. Thus, although the pattern in the noun response window showed differences when analyzing within-language and cross-language conditions in isolation, the difference was not substantial enough to become significant during the between-experiment analyses. The between-experiment comparison indicates that the processing of lexical representations associated with disconfirmed predictions follows a similar pattern across the two language contexts. This finding contrasts with the conclusions reported in Liao and Chan (2016) who showed the influence of language switching on prediction. However, this apparent difference likely stems from a distinct research focus: the present study investigated the time course of eye movements associated with words that were predicted but not ultimately presented (i.e., disconfirmed predictions), whereas Liao and Chan (2016) examined ERP responses to actual incoming linguistic input. Therefore, in contrast to Liao and Chan (2016), who found an interaction between semantic predictability and language switching, our results suggest that the observed fixation patterns associated with disconfirmed predictions are broadly similar across within-language and cross-language contexts. If these fixation patterns reflect residual activation and subsequent inhibition, such activation patterns do not appear to differ substantially between the two language contexts.

Such interpretations are plausible in light of the evidence from two additional studies. To elaborate, the first supporting evidence comes from a very recent study (Yin & Pickering, 2026). In their study, the researchers found that when Chinese-English participants generated a prediction for upcoming English target words in highly constraining English contexts, they also increased their fixations on pictures whose Chinese names were homophones of the Chinese translations of those predictable English words. This finding clearly supports a language-non-selective prediction in a two-language context. Thus, the residual activation observed immediately after prediction disconfirmation should be similar in both cross-language and within-language contexts. The second supporting study, by Sánchez-Meléndez and colleagues (2024), suggested that a domain-general inhibitory control mechanism may be recruited during the suppression of a disconfirmed prediction. This suggests that, if an inhibitory process operates at a later stage of target noun processing in our current study, suppressing a predicted but subsequently disconfirmed word may rely on a domain-general mechanism that functions independently of the language relationship between the input and the preceding context.

However, some limitations of the current study also need to be mentioned. First, although we ensured that the nouns within each trial were either all semantically related or all relatively unrelated, thus preventing any single noun/picture from standing out, some differences in visual salience may nevertheless remain, as it is inherently difficult to fully control both visual salience and semantic congruency to the classifier across the three images in each trial. Yet, given that participants had time to prescreen the pictures before the classifier and noun target were presented, we believe these effects to be negligible (Apfelbaum et al., 2021). Second, as our primary focus concerning classifier-noun congruency was on a meaningful and natural association between the classifier and the noun, we cannot distinguish between semantic and grammatical properties of Mandarin Chinese classifiers (cf. Grüter et al., 2020). That is, for the type of Chinese classifiers we used semantic (in)congruence also coincided with grammatical (in)congruence: classifier–noun pairs in the congruent condition were both semantically and grammatically congruent, whereas those in the incongruent condition were semantically and grammatically incongruent (please note that with respect to English grammatical congruence concerning the singular/plural form of nouns, classifiers and nouns were always grammatically congruent to avoid additional influences). In future research it would thus be interesting to disentangle semantic and grammatical influences of Chinese classifiers by including different types of classifiers. Third, as the present study focused exclusively on prediction generated during L1 comprehension, further empirical work is necessary to establish whether disconfirmed predictions generated during L2 comprehension exhibit the same pattern of language-independent activation and subsequent inhibition. Fourth and finally, an additional limitation concerns the interpretation of eye movements as an index of underlying cognitive processes. Although the VWP provides a sensitive measure of the time course of language processing and has a long history of being used to study activation patterns during language processing (e.g., Huettig et al., 2011; Roembke & McMurray, 2025), fixation patterns are influenced not only by lexical processing but also by the dynamics of eye-movement generation, including saccade planning and fixation duration (McMurray, 2023). Consequently, the present findings should be interpreted as reflecting patterns of eye movements that may be associated with sustained activation and subsequent inhibition, rather than as providing a direct measure of these cognitive mechanisms.

In conclusion, the present study demonstrates a temporal pattern of eye movements that is compatible with residual activation of disconfirmed predictions shortly after disconfirmation and may also reflect subsequent inhibitory processes in both within-language and cross-language contexts. These findings suggest a dynamic trajectory in the processing of disconfirmed predictions. Furthermore, this processing pattern appears to be comparable across within-language and cross-language contexts.

## Additional File

The additional file for this article can be found as follows:

10.5334/joc.518.s1 Appendix A. Classifier-noun pairs used in Experiment 1 and Experiment 2. Congruent words are bolded.

## Data Availability

The data and analysis code that support the findings of this study are openly available at https://osf.io/g694j/.

## Footnotes

The duration of the auditory noun presentation varied depending on the number of syllables the noun had. The mean duration of the noun presentation was 778 ms (SD = 89 ms). An additional 2 ms was added to make the time-window duration of the two experiments comparable, resulting in a total noun presentation duration of 780 ms for the analysis.

The duration of the auditory noun presentation varied depending on the number of syllables the noun had. The mean duration of the noun presentation was 777 ms (SD = 111 ms). An additional 3 ms was added to make the time-window duration of the two experiments comparable, resulting in a total noun presentation duration of 780 ms for the analysis.
