# Supplementary material for: Prediction During Within- and Cross-Language Phrase Comprehension: Evidence from Eye-Tracking
Source: J Cogn. 2026 Sep 21;9(1):47. doi: 10.5334/joc.518 (PMC13614251; doi:10.5334/joc.518)
Supplement: Supplementary file 1 [file joc-9-1-518-s1.pdf]

Appendix A

Classifier-noun pairs used in Experiment 1 and Experiment 2. Congruent words are bolded.

Classifier	Picture 1	Picture 2	Picture 3	Condition
a barrel of<br>一桶	biscuits<br>饼干	beef<br>牛肉	olives<br>橄榄	none-congruent-picture
	**wine**<br>**葡萄酒**	chocolates<br>巧克力	ham<br>火腿	one-congruent-picture
	**oil**<br>**石油**	**beer**<br>**啤酒**	girls<br>女孩	two-congruent-pictures
a basket of<br>一篮	birds<br>鸟	earrings<br>耳环	cash<br>现金	none-congruent-picture
	**apples**<br>**苹果**	yogurt<br>酸奶	pills<br>药丸	one-congruent-picture
	**eggs**<br>**鸡蛋**	**flowers**<br>**鲜花**	chopsticks<br>筷子	two-congruent-pictures
a bottle of<br>一瓶	hay<br>干草	donuts<br>甜甜圈	chess<br>棋	none-congruent-picture
	**milk**<br>**牛奶**	**pills**<br>药丸	oranges<br>橙子	two-congruent-pictures
	**perfume**	**juice**	wood	two-congruent-pictures

	**香水**	**果汁**	木头	
a bowl of	furniture	ropes	tissues	none-congruent-picture
一碗	家具	绳子	纸巾	
	**noodles**	firemen	crayons	one-congruent-picture
	**面条**	消防员	蜡笔	
	**soup**	ribbons	toothpaste	one-congruent-picture
	**汤**	丝带	牙膏	
a box of	firemen	boys	doctors	none-congruent-picture
一盒	消防员	男孩	医生	
	**matches**	**cereals**	bricks	two-congruent-pictures
	**火柴**	**麦片**	砖头	
	**donuts**	**crayons**	birds	two-congruent-pictures
	**甜甜圈**	**蜡笔**	鸟	
a bundle of	juice	rain	water	none-congruent-picture
一捆	果汁	雨	水	
	**hay**	candies	snow	one-congruent-picture
	**干草**	糖果	雪	
	**newspapers**	**wood**	oil	two-congruent-pictures
	**报纸**	**木头**	石油	
a coil of	fish	chocolates	ice	none-congruent-picture
一卷	鱼	巧克力	冰	

	**rope**	ink	eggs	one-congruent-picture
	绳子	墨水	鸡蛋	
	**wire**	**ribbon**	butter	two-congruent-pictures
	电线	丝带	黄油	
a crew of	ham	corns	eggs	none-congruent-picture
一队	火腿	玉米	鸡蛋	
	**firemen**	cereals	wire	one-congruent-picture
	消防员	麦片	电线	
	**doctors**	matches	bread	one-congruent-picture
	医生	火柴	面包	
a cube of	hand	noodles	flower	none-congruent-picture
一块	手	面条	鲜花	
	**ice**	knife	glue	one-congruent-picture
	冰	刀	胶水	
	**candy**	**butter**	flour	two-congruent-pictures
	糖果	黄油	面粉	
a cup of	paper	knives	wire	none-congruent-picture
一杯	纸	刀	电线	
	**tea**	noodles	beef	one-congruent-picture
	茶	面条	牛肉	
	**water**	hands	earrings	one-congruent-picture

	**水**	**手**	**耳**环	
a dish of	books	bricks	lipsticks	none-congruent-picture
**一**盘	书	砖头	**口**红	
	**fish**	**chips**	tea	two-congruent-pictures
	**鱼**	**薯条**	**茶**	
	**beef**	**ice-cream**	juice	two-congruent-pictures
	**牛肉**	**冰激凌**	**果汁**	
a drop of	soil	bread	toothpaste	none-congruent-picture
**一滴**	**土**	**面包**	**牙膏**	
	**rain**	wheat	book	one-congruent-picture
	**雨**	**麦子**	书	
	**ink**	biscuit	chess	one-congruent-picture
	**墨水**	饼干	**棋**	
a grain of	clothes	cigarette	plate	none-congruent-picture
**一粒**	**衣服**	**烟**	盘子	
	**rice**	furniture	newspaper	one-congruent-picture
	**米**	**家具**	报纸	
	**wheat**	**corn**	donut	two-congruent-pictures
	**麦子**	**玉米**	**甜甜圈**	
a group of	peanuts	beans	cereals	none-congruent-picture
**一群**	**花生**	**豆子**	**麦片**	

	**girls**	cash	beans	one-congruent-picture
	女孩	现金	豆子	
	**boys**	ice	coins	one-congruent-picture
	男孩	冰	硬币	
a handful of	milk	flour	jam	none-congruent-picture
一把	牛奶	面粉	果酱	
	**peanuts**	honey	beer	one-congruent-picture
	花生	蜂蜜	啤酒	
	**beans**	**grapes**	soup	two-congruent-pictures
	豆子	葡萄	汤	
a jar of	shoes	newspaper	ribbons	none-congruent-picture
一罐	鞋	报纸	丝带	
	**jam**	clothes	plates	one-congruent-picture
	果酱	衣服	盘子	
	**olives**	lipsticks	stamps	one-congruent-picture
	橄榄	口红	邮票	
a nest of	butter	oranges	ice-cream	none-congruent-picture
一窝	黄油	橙子	冰激凌	
	**birds**	perfume	cheese	one-congruent-picture
	鸟	香水	奶酪	

	**ants**	peanuts	shoes	one-congruent-picture
	蚂蚁	花生	鞋子	
a packet of	snow	ink	girls	none-congruent-picture
一包	雪	墨水	女孩	
	**cigarettes**	**biscuits**	wine	two-congruent-pictures
	烟	饼干	电线	
	**tissues**	**chocolates**	ants	two-congruent-pictures
	纸巾	巧克力	蚂蚁	
a pair of	chips	wheat	honey	none-congruent-picture
一双	薯条	麦子	蜂蜜	
	**earrings**	**hands**	soil	two-congruent-pictures
	耳环	手	土	
	**chopsticks**	**shoes**	rice	two-congruent-pictures
	筷子	鞋	米	
a pile of	soup	oil	wine	none-congruent-picture
一堆	汤	石油	葡萄酒	
	**paper**	jam	boys	one-congruent-picture
	纸	果酱	男孩	
	**bricks**	**clothes**	rain	two-congruent-pictures
	砖头	衣服	雨	
a set of	rice	grapes	yogurt	none-congruent-picture

一套	米	葡萄	酸奶	
	**chess**	**knives**	doctors	two-congruent-pictures
	**棋**	**刀**	医生	
	**furniture**	**stamps**	apples	two-congruent-pictures
	**家具**	**邮票**	苹果	
a shovel of	candies	tea	cheese	none-congruent-picture
一铲	糖果	茶	奶酪	
	**snow**	corns	ropes	one-congruent-picture
	**雪**	玉米	绳子	
	**soil**	paper	grapes	one-congruent-picture
	**土**	纸	葡萄	
a slice of	pill	glue	crayon	none-congruent-picture
一片	药丸	胶水	蜡笔	
	**bread**	**orange**	chip	two-congruent-pictures
	**面包**	**橙子**	薯条	
	**ham**	**cheese**	ice-cream	two-congruent-pictures
	**火腿**	**奶酪**	冰激凌	
a spoonful of	matches	stamps	chopsticks	none-congruent-picture
一勺	火柴	邮票	筷子	
	**yogurt**	tissues	flowers	one-congruent-picture
	**酸奶**	纸巾	鲜花	

	**honey**	**flour**	cigarettes	two-congruent-pictures
	**蜂蜜**	**面粉**	烟	
a stack of	perfume	beer	ants	none-congruent-picture
一摞	香水	啤酒	蚂蚁	
	**coins**	**plates**	water	two-congruent-pictures
	**硬币**	**盘子**	水	
	**cash**	**books**	milk	two-congruent-pictures
	**现金**	**书**	牛奶	
a tube of	apples	coins	wood	none-congruent-picture
一管	苹果	硬币	木头	
	**lipstick**	fish	hay	one-congruent-picture
	**口红**	鱼	干草	
	**toothpaste**	**glue**	olives	two-congruent-pictures
	**牙膏**	**胶水**	橄榄	
